# Solvent Effect on the Self-Assembly of a Thin Film Consisting of Y-Shaped Copolymer

**DOI:** 10.3390/polym11020261

**Published:** 2019-02-04

**Authors:** Dan Mu, Jian-Quan Li, Xing-Shun Cong, Yu-Wei Mi, Han Zhang

**Affiliations:** 1 College of Chemistry Chemical Engineering and Materials Science, Zaozhuang University, Zaozhuang 277160, China; congxs@uzz.edu.cn (X.-S.C.); miyw@uzz.edu.cn (Y.-W.M.); zhangh@uzz.edu.cn (H.Z.); 2 Advanced Photonics Center, Southeast University, 2# Sipailou, Nanjing 210096, China; 3 Opto-Electronic Engineering College, Zaozhuang University, Zaozhuang 277160, China; lijq786@163.com

**Keywords:** solvent effect, copolymer, self-assembly, confinement, thin film

## Abstract

The self-assembly of an amphiphilic Y-shaped copolymer consisting of two hydrophilic branches and one hydrophobic branch in a thin film is investigated under different conditions by virtue of mesoscopic computer modelling, accompanied by doping with a single solvent, doping with a binary solvent, and those solvent environments together with the introduction of confinement defined by various acting distances and influencing regions. A cylindrical micellar structure is maintained, as it is in the thin film with the doping of either 10% hydrophobic solvent or 10% hydrophilic solvent, whose structure consists of the hydrophobic core and hydrophilic shell. Attributed to the hydrophobicity/hydrophilia nature of the solvents, different solvents play an obvious role on the self-assembled structure, i.e., the hydrophobic solvent presents as a swelling effect, conversely, the hydrophilic solvent presents as a shrinking effect. Further, the synergistic effect of the binary solvents on the self-assembly produces the lowest values in both the average volumetric size and free energy density when the quantity of hydrophobic solvent and hydrophilic solvent is equivalent. Interestingly, the solvent effect becomes more pronounced under the existent of a confinement. When a lateral-oriented confinement is introduced, a periodically fluctuating change in the cylindrical size occurs in two near-wall regions, but the further addition of either hydrophobic or hydrophilic solvent can effectively eliminate such resulting hierarchical-sized cylinders and generate uniform small-sized cylinders. However, with the introduction of a horizontal-orientated confinement, the copolymers self-assemble into the spherical micellar structure. Moreover, the further addition of hydrophobic solvent leads to a decrease in the average size of micelles via coalescence mechanism, in contrast, the further addition of hydrophilic solvent causes an increase in the average size of micelles via splitting mechanism. These findings enrich our knowledge of the potential for the solvent effect on the self-assembly of amphiphilic copolymer system, and then provide theoretical supports on improving and regulating the mesoscopic structure of nanomaterials.

## 1. Introduction

Due to their abundance in thermodynamics and kinetics [1,2], as well as their wide use as an industrial commodity in coatings and lubricants [3,4,5], polymer thin films (thickness less than 100 nm) have become an indispensable part of the developmental needs in modern high-tech applications such as nanolithography, biotechnology, optoelectronics, novel sensors and actuators [6,7,8,9,10,11]. The investigation of thin polymer films has experienced rapid growth, involving research in physics, chemistry, materials science, polymer science, engineering, and even clinical science. In contrast to other construction materials for polymer thin films, block copolymers offer an efficient and impressive bottom-up route to generate a variety of well-defined nanoscopic structures, including spheres, cylinders and lamellae, which are potentially applicable to nanofabrication applications for photovoltaic cells [12], nanoparticle templates [13], low-*k* dielectrics [14], nanostructured membranes [15], high-density data storage media [16], etc. Numerous studies have revealed that the scale and spatial symmetry of self-assembled structures depend on many factors concerning the block copolymers, including the grafting density or chain length, the relative content of the constituting blocks or the segment length, and the grafting sequence or distribution of the grafting points [17,18,19,20,21,22,23,24]. Among a large variety of copolymers, amphiphilic copolymers composed of hydrophilic and hydrophobic components are promising and useful candidates in a rich variety of applications, such as chiral separation membranes [25], drug release systems [26], antifouling coatings [27], gas and biosensors [28], and activating carriers for biocatalysts [29]. To advance the properties or to meet the requirements of device functionality, the aggregating structure or orientation at the mesoscopic scale are controlled by methodologies including the introduction of thermal annealing [30], external fields [31,32], solvent annealing [33], shear [34,35] and patterned substrates [36,37,38,39,40,41,42,43,44,45,46,47,48].

In our previous work [49], the self-assembly of a reformed symmetric H-shaped amphiphilic copolymer consisting of four hydrophilic branches and one hydrophobic stem was systematically investigated. From observations after mesoscopic simulation, it was proven that the existence of vacancies is vital to regulate the sizes of self-assembled cylindrical micelle structures. Thus, in this study, a further-reformed Y-shaped amphiphilic copolymer is the new research object, whose molecular topology is obtained by tailoring the H-shaped amphiphilic copolymer from the middle of the hydrophobic stem. Both are symmetric molecular structures. Compared with the H-shaped copolymer, the Y-shaped copolymer possesses a reduction of hydrophilia by containing half of the number of the hydrophilic branches of the H-shaped polymer, inspiring our interest in its self-assembled structure in the thin film only and under confinement. To date, the presence of selective solvents was observed to influence self-assembly through experimental and theoretical methods [50,51,52]. Therefore, an intensive investigation of the solvent effect on self-assembly in response to different external conditions (according to the conditions of being in the thin film only and being under confinement, as mentioned above) is also exerted. Herein, the introduced solvent environment involves three situations, i.e., a hydrophobic or hydrophilic single selective solvent and a binary solvent constituted of both hydrophobic and hydrophilic solvents mixed in a certain proportion. In addition to the comparison of self-assembly between the Y-shaped copolymer system and the H-shaped copolymer system, which can exhibit the features of the Y-shaped copolymer system, different solvent effects on the self-assembly are also discussed, which are expected to elucidate the according mechanism, aiming to provide reliable theoretical evidence for preparing well-defined materials via regulation of the external factors.

## 2. Models and Parameter Settings 

Compared with atomistic calculations or simulation methods, the mesoscopic modeling method is potent for examining the mesoscale properties of materials, providing a solid bridge between fast molecular kinetics and slow thermodynamic relaxation and facilitating the understanding of structure-property relationships. In particular, the input parameters of mesoscopic simulations always originate from experimental data or the results of atomistic calculations, which guarantee the validity and rationality of in-process results and achieve reasonable final results. Among the available mesoscopic dynamic methods, MesoDyn has been validated as an effective approach to explore the self-assembly behavior of polymer systems at the mesoscale [53,54]. The intrinsic theory of the MesoDyn method is based on the dynamic mean-field theory developed by Fraaije [55,56]. Herein, the Langevin equations are used to describe the phase separation dynamics and ordering processes of the polymeric systems, and the Gaussian chain density functionals are established by one-to-one correspondence between the external potential fields and the density fields for each bead type. Furthermore, the intrinsic chemical potentials are the functionals of external potentials and density fields. Hence, the coupled Langevin equations can describe the relationship between the time derivatives and the intrinsic chemical potentials. Due to the association between the noise source and the exchange kinetic coefficients, a closed set is formed by these equations, which is efficiently integrated into a cubic mesh by a Crank–Nicholson scheme for iteration of the inner loop [57]. If all beads are of the same size for simplicity, then the chain topology primarily depends on the coarse-graining of the atomic model.

**Scheme 1:**
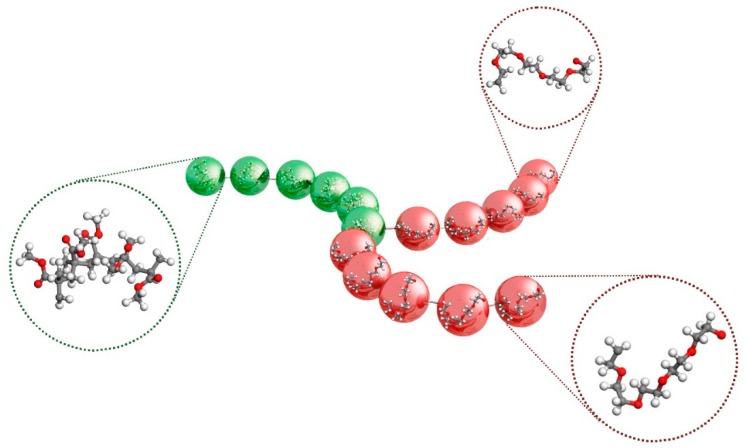
Schematic representation of the Y-shaped copolymer. The red particles representing the coarse-grained EO blocks are denoted A, and the green particles representing the coarse-grained MMA blocks are denoted B. Three molecular structures in three individual beads are amplified in dotted circles; herein, red, grey and white balls denote oxygen, carbon and hydrogen atoms, respectively.

The coarse-grained mapping between the atomistic and mesoscopic model of the poly(ethylene oxide)-*b*-poly(methyl methacrylate) (PEO-*b*-PMMA) copolymer involves reconstruction of the molecular structure and conversion of the interaction parameters, as elaborated in our previous study [58]. First, eight PEO homopolymers with incremental chain lengths of 10, 20, 30, 40, 50, 60, 70 and 80 were built on the basis of the rotation isomeric state (RIS) model of Flory. To reduce the possible presence of thermodynamic hot spots in the models, a subsequent cycled treatment of molecular mechanics and molecular dynamics was executed on the constructed PEO homopolymer atomic models, and then, the corresponding solubility parameters were determined independently. After analyzing the change trends of the eight solubility parameter values with their corresponding chain lengths, we found that the calculated solubility parameters for chain lengths equal to 50 and greater than 50 repeating units are all close to the experimental value [59]. For economic calculation, the minimum representative polymer chain length of PEO was confirmed to be 50, whose solubility parameter was the threshold for maintaining a relatively steady value for PEO homopolymers with chain lengths equal to and longer than 50. For the other constituent of the PEO-*b*-PMMA copolymer, PMMA, the treatment procedures for determining the minimum representative polymer chain length of PMMA homopolymers followed the same routine as that used for the PEO homopolymers and resulted in a length of 50, and the solubility parameter met a reasonable tolerance in the scope of the experimental value [59]. The coarse-grained molecular structures of the original atomic PEO_50_ and PMMA_50_ homopolymers were verified via their minimum representative polymer chain lengths divided by their individual characteristic ratios of *C*_∞_(PEO) = 9.89 and *C*_∞_(PMMA) = 8.65 obtained from the Polymer Handbook [60], resulting in A_5_ (A bead denotes the EO component) and B_6_ (B bead denotes the MMA component), respectively. Hence, a combined Y-shaped amphiphilic copolymer mesoscopic model is constructed from two A_5_ segments as the hydrophilic branches and one B_6_ segment as the hydrophobic branch, with a common joint to the B bead, as shown in Scheme 1. The connection in the pairwise interaction energy between the microscale and mesoscale is as follows:

*E*_ij_ = *χ*_AB_*RT* (1)

where the Flory–Huggins interaction parameter, *χ*_AB_, is calculated by atomistic simulations of various compositions, *R* is the molar gas constant, 8.314 J·mol^−1^·K^−1^, and *T* is 400 K, with the calculated *χ* values being positive at this temperature. *E*_ij_ is the input parameter used in the MesoDyn simulation to describe the pairwise interaction between A and B beads. Positive values of the interaction parameters indicate repulsive interactions between pairwise components, which can enhance microseparation in the copolymer system. According to the difference in the hydrophilicity of the EO and MMA components, including two types of solvents, i.e., hydrophilic and hydrophobic solvents, all the interaction parameters are listed in Table 1.

The same *L_x_* × *L_y_* × *L_z_* spatial lattice with a 32 × 32 × 5 nm^3^ grid is adopted to model the thin film in all mesoscopic simulations. Here, 5 nm, which is only 15.6% of the 32-nm width, corresponds to the thickness of the thin film. To ensure the isotropy of all grid-restricted operators, the bond length is set to be 1.1543. Neglecting the size influence on self-assembly for simplicity, all bead sizes are 0.01 nm^3^, and the bead diffusion coefficient, which provides the link between the real time scale of the simulation and the dimensionless units used by the MesoDyn simulation engine, is accepted with the default value of 1.0 × 10^−7^ cm^2^·s^−1^. Moreover, owing to the inverse relationship of the dimensionless noise parameter to the level of thermal noise applied, the maximum value of 100 for the noise-scaling parameter is used to realize the least noise. Owing to different situation for various systems, the non-confined systems are in periodic boundary conditions, but the confined systems are in non-periodic boundary condition with the introduction of the confinements. Additionally, the compressibility parameter is fixed at 10.0, and the dimensionless time step is set to be 0.5. The simulation of each system is conducted for 4.0 × 10^5^ steps. 

## 3. Results and Discussion

### 3.1. Self-Assembled Structure in the Single Solvent

**Figure 1:**
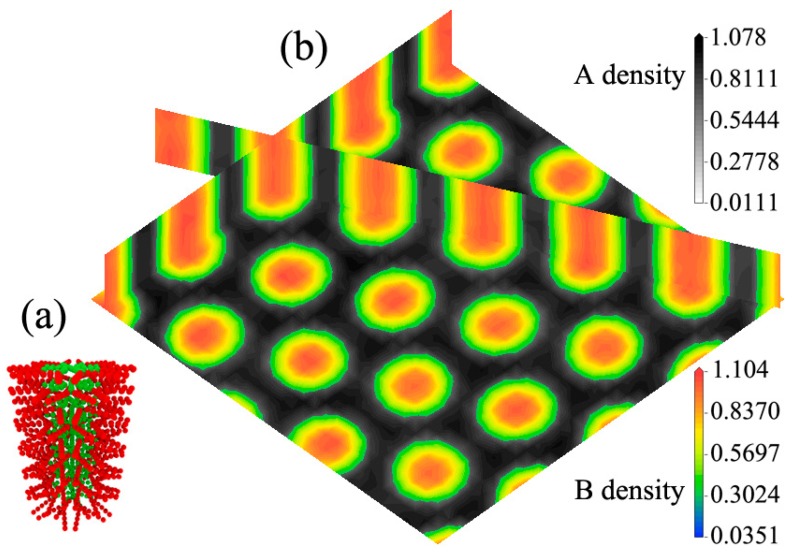
(**a**) Illustration of a cylindrical self-assembly structure formed by Y-shaped copolymer. The red particles are denoted A beads, and the green particles are denoted B beads. (**b**) One density slice along the X–Y plane, and two density slices through the cylindrical axes in two cylindrical arrays, where the latter two are vertical to the former one. The color mappings of A and B beads are in white-black and reverse rainbow, respectively.

**Figure 2:**
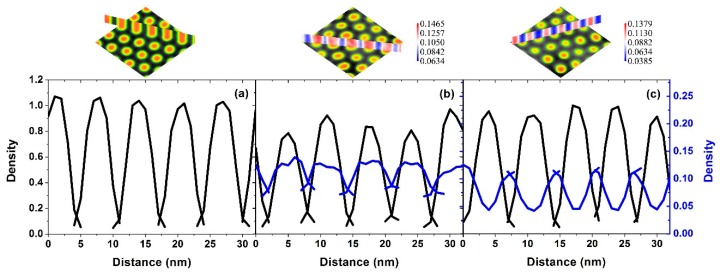
Density distributions of B beads (denoted by black line) and solvent beads (denoted by blue line) from the radial direction of a self-assembled cylinder array in (**a**) plain copolymer; (**b**) copolymer doped with 10% hydrophobic solvent; and (**c**) copolymer doped with 10% hydrophilic solvent systems. Three density-slice pictures are placed above each subfigure, respectively. In each density-slice picture, one density slice lays along the X–Y plane, and the other density slice goes through the cylindrical axes in the same cylindrical array, where the latter is vertical to the former. The color mappings of A, B and solvent beads are in white-black, reverse rainbow and blue-white-red, respectively.

Such construction with two ends, both consisting of hydrophilic branches, and the third end being formed by a hydrophobic branch guarantees that the short-chained copolymer molecules will encounter less steric hindrance and entanglement during the self-assembly process. Additionally, compared with the broad space in the XY plane, the relatively narrow space along the Z axis makes the conformations of the copolymeric molecules very monotonous, possessing an extended conformation in the XY plane but being largely flattened along the Z axis. Moreover, the constituent content of hydrophilic branches for each copolymer is relatively high, so the possibility of “meeting with” the same component is higher for the EO component than for the MMA component, which results in the outer coalescence of the EO component facilitating acceleration of the formation of the inner hydrophobic core in the self-assembled structure, and then, a core-shell-structured plane is formed. Driven by aggregation of the same components, the core-shell-structured planes adopt a layer-by-layer stacking mode in the direction of the film thickness and then self-assemble into a core-shell-structured cylinder, as shown in Figure 1a, which is consistent with experimental observations of the hydrophobic components self-assembled into the core portion via stacking the ‘disks’ from both the W. Jiang group [61] and the A. Hillmyer group [62]. Subsequently, the cylindrical self-assembled structure with the hydrophilic shell surrounding the hydrophobic core comes into being. Similarly, the H-shaped copolymer also self-assembles into the same kind of core-shell cylindrical micelle structure. Compared with the H-shaped copolymer [49], the Y-shaped copolymer lacks two hydrophilic branches, facilitating the monomers being relatively flexible in adjusting their conformations to generate a relatively shrunken conformation in the XY plane during the self-assembly process, resulting in a 22.84% decrease in the average cylinder size. Additionally, the condensed aggregating domains inhibit the formation of vacancies, so the fluctuating span of the average size of cylinders over time evolution is much narrower, resulting in a quite similar component distribution in the assembled cylinders, and the final self-assembled structure is shown in Figure 1b, which is proven by quantitative analysis of the density distribution of B beads shown in Figure 2a. For each assembled cylinder, whose left-branch of density distribution can nearly overlap that of the right-branch, indicates a homogeneous allocation of the copolymer molecules in each cylinder and can be regarded as evidence of the layer-by-layer assembly mode. Furthermore, the almost identical density distribution of the five cylinders from the same array manifests a uniform and ordered arrangement and declares the promising application of such a Y-shaped amphiphilic copolymer to generate highly ordered self-assembled structures.

From the density distribution of B beads shown in Figure 2b,c, we see that the self-assembled cylindrical structure of the copolymer remains unchanged, even when doped with solvents of different hydrophilicities. However, in contrast to the high similarity between the density distribution for the five cylinders in Figure 2a, such similarity obviously decreases with introduction of the solvent effect, for which the quantitative data are listed in Table 2. This effect is particularly remarkable for the copolymer system doped with the hydrophobic solvent, in which the solvent repulsive interaction with A beads is stronger than that with B beads, which is beneficial to pushing the hydrophobic solvent beads to pack into the core domain of the cylinder, as shown in Figure 2b. The small volume of the solvent molecules facilitates rapid coalescence of the solvent beads into a broader density distribution, with a larger coverage area in the core domain than that around the shell domain. Aside from the influx of hydrophobic solvent beads into the inner self-assembled structure, whose extra interaction increases the freedom of motion of the hydrophobic branches, the resultant uneven distribution of the solvent beads can also reduce the ordered arrangement of the hydrophobic branches, finally resulting in the highest standard deviation of 0.080, which is 3.63-fold higher than the lowest value of 0.022, indicating an evident inhomogeneous density distribution of B beads in the different self-assembled cylinders.

In contrast, the repulsive interaction of hydrophilic solvent beads is stronger with B beads than with A beads, so most of the solvent beads cannot access the central zone of the core domain of the self-assembled cylinders and can only aggregate around the shell domain of cylinders, as shown in Figure 2c. This leads to less influence from the solvent beads on the conformation and arrangement of copolymers in the core domain of the cylinders. A small decrease of 8.24% in the average peak value of B beads indicates that the solvent beads limitedly affect the copolymer arrangement in the internal region of the assembled cylinders, but delivery of the influence from the external region of the assembled cylinders to change the conformation of the copolymer will overwhelmingly attenuate when it reaches the central zone of the core domain of the assembled cylinders. Additionally, such small and low-quantity solvent beads are unfavorable in forming the same type of “solvent shell” outside each cylinder. Compared with the plain copolymer system, the small increase of 0.017 in the standard deviation upon doping with 10% hydrophobic solvent reveals the slight influence of the solvent beads on changing the copolymer arrangement in each cylinder, which agrees with the experimental study of J. W. Jeong et al., which stated that in the hydrophilic component of selective solvent vapors, cylinders are expected to be the more stable morphology [33].

**Figure 3:**
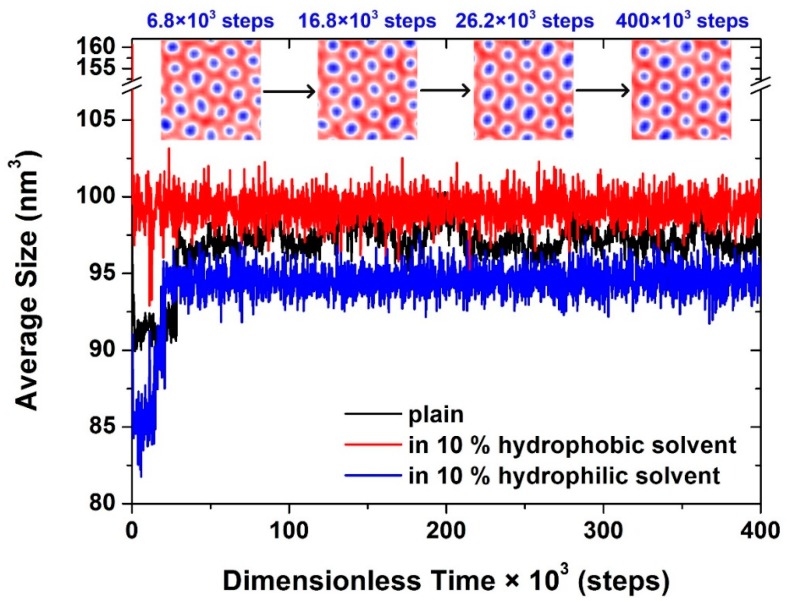
Evolution of the average size of the cylindrical structure with simulation time in three different systems, i.e., the plain system, the system doped with 10% hydrophobic solvent and the system doped with 10% hydrophilic solvent denoted by black, red and blue lines, respectively. The pictures for the hydrophilic solvent beads are displayed above at representative time, whose color mapping is in blue-white-red.

In addition to the above static analysis of the final structure formed under different circumstances, a dynamic analysis of the average volumetric size of the self-assembled cylinders during the entire simulation, shown in Figure 3, is also needed and is a helpful route by which to understand the evolution of the cylindrical structure and the involved solvent effect. In the plain copolymer system, the initial homogeneous state lasts for less than 50 steps. A bulky aggregate is formed by random gathering of the copolymers, which is rapidly broken into several pieces of aggregates. Over 26.2 × 10^3^ steps, the copolymers refine their conformations and distributions in individual aggregate, resulting in a quasi-cylindrical structure. Driven by the higher surface free energy density of the small aggregates, proper coalescence of these aggregates reduces the free energy density of the system. Later, 2.2 × 10^3^ steps are required for the system to reach a stable thermodynamic state. The remaining time is the second period for the copolymer optimization of the coalesced aggregates into regular cylinders, so that the self-assembly of copolymers into cylindrical structures is accomplished. However, the introduction of hydrophobic solvent beads, which are present in a much smaller amount than is the copolymer, presents a weaker repulsive interaction with the constituents in the core domain of the cylinder than that with the constituents in the outer corona, which facilitates entry of the solvent beads into the core domain of the cylinder. Under quick invasion of the solvent, both solvent occupation of the space and its weak repulsion of the MMA component lead to the internal copolymers being squeezed out to some extent, which causes an expansion of the cylinder to a larger size than that of the plain system. However, the change tendencies of the average size of the cylinders are quite similar between the system with the doped hydrophilic solvent and the plain copolymer system. They involve the formation of a bulky aggregate, breakage into small aggregates, fusion of the small aggregates into larger ones, and optimization of the cylindrical structure. Two distinct differences are found with the introduction of the hydrophilic solvent, i.e., (1) instead of one, there are two successive stages of fusing the small aggregates and (2) the average size of the final formed self-assembled cylinders is smaller than that in the plain system. These distinctions are derived from the introduction of the hydrophilic solvent, which possesses a weaker repulsive interaction with the EO component than with the MMA component, causing most of the solvent beads to gather around the core domain as a “solvent shell”. Thus, the blending of small solvent beads into the foremost bulky aggregate is beneficial to breaking it into small aggregates, which present an average size 6.39% smaller than that of the aggregates in the plain system at the same stage. The newly formed outside shell of solvent beads has a small size that avails sequential opening and fusing of the shells. The subsequent stage of coalescence is expedited, and another coalescence stage immediately follows, resulting in a 4.42 nm^3^ and 4.72 nm^3^ increase in the average size, respectively. Such a sped-up function leads to the two coalescence stages being reduced by 7.0 × 10^3^ steps compared with that of the plain system. Furthermore, the “wrapped outside” solvent shell compels the hydrophilic branches to adopt a contracted conformation. Accordingly, the conserved conformational energy of the EO component is released via transfer from the contracted conformation inside to the hydrophobic branches, resulting in a decrease of 2.44 nm^3^ in the average size of the aggregates.

### 3.2. Self-Assembled Structure in the Binary Solvent

**Figure 4:**
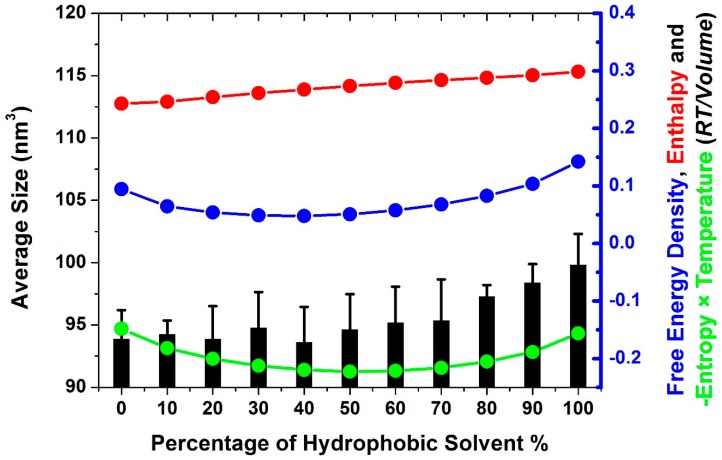
Average size, free energy density, enthalpy and -entropy × temperature in systems with different doping percentages of hydrophobic solvent, which are denoted by a black column, blue line, red line, and green line, respectively.

In different types of single-solvent environments, regardless of hydrophobic or hydrophilic nature, based on static analysis of the self-assembly structure and dynamic analysis of the evolution of the average volumetric size of the assembled cylinders, the solvent definitely plays a significant role in influencing the self-assembled structure. As a matter of course, the investigation of self-assembly in binary solvents is essential. To facilitate comparable analysis with the single-solvent doping system, the total amount of introduced hydrophilic and hydrophobic solvent is still 10%. Nine copolymer systems doped with a binary solvent in mixing ratios of hydrophobic solvent to the total solvent of 10%, 20%, 30%, 40%, 50%, 60%, 70%, 80% and 90% are investigated. Moreover, two additional copolymer systems doped with only hydrophilic solvent or hydrophobic solvent are added as references in this investigation. To maintain consistency with the above nine systems in the description of the binary solvents, these two reference systems are equivalent to the mixing ratios of hydrophobic solvent of 0% and 100%, respectively. The related structure (i.e., average size) and thermodynamic data (i.e., free energy density and enthalpy) of these eleven systems are shown in Figure 4. With the increase in the amount of hydrophobic solvent, the enthalpy increases almost in direct proportion, whereas the free energy density first decreases and then increases, and the notable turning point is at the copolymer system doped with 40% hydrophobic solvent. The lowest average size appears at this turning point, which separates the change in free energy density into two different change tendencies. Before the turning point, there are a smaller average size and a decreasing trend; however, after the turning point, a larger average size occurs and an increasing trend is presented.

**Figure 5:**
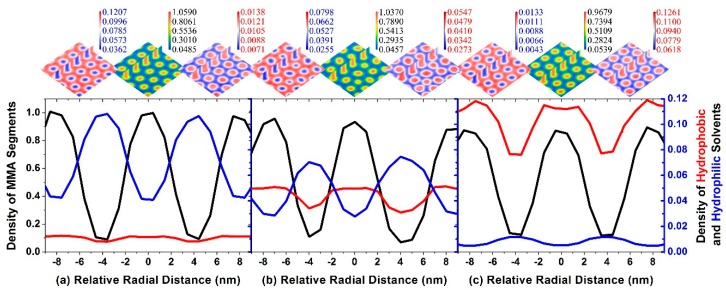
Density distributions of B beads (denoted with a black line), hydrophobic solvent beads (denoted with a red line) and hydrophilic solvent beads (denoted with a blue line) in the radial direction of self-assembled cylinder arrays in three systems doped with binary solvent, corresponding to (**a**) the system doped with 10% hydrophobic solvent; (**b**) the system doped with 40% hydrophobic solvent; and (**c**) the system doped with 90% hydrophobic solvent. The density distributions of three types of beads along the thickness direction of the thin film are omitted for concise display. Three density-slice pictures are exhibited above each subfigure. They are pictures for the hydrophilic solvent beads (whose color mapping is in blue-white-red), the B beads (whose color mapping is in reverse rainbow) and the hydrophobic solvent beads (whose color mapping is in blue-white-red) from left to right, respectively. Additionally, for clarity, the captions are colored the same as they are in the corresponding density-distribution lines.

To deeply explore self-assembly in binary solvent, a structural analysis of three representative systems is performed, for which the results are shown in Figure 5. The systems are doped with 10%, 40% and 90% hydrophobic solvent, representing the system with the most hydrophilic solvent, the system at the turning point, and the system with the most hydrophobic solvent, respectively. In the first system, shown in Figure 5a, the small amount of hydrophobic solvent beads distributes almost equally and exerts a limited influence on the system. In contrast, with a large amount of hydrophilic solvent beads gathering around the MMA-rich region of the cylinder, the repulsive force between the hydrophilic solvent shell and the inside MMA component causes shrinkage of the hydrophobic branches of the copolymers towards the inside, resulting in a comparatively small average size of the assembled cylinders. When the hydrophobic solvent content is increased to 40%, theoretically speaking, two solvent shells are formed, one is the hydrophobic solvent shell around the corona domain of the cylinder, and the other is the hydrophilic solvent shell around the hydrophobic core of the cylinder. In fact, the results for nearly equal contents of the two solvents show that there is no obvious boundary between the two solvent shells. They form a mixed solvent environment, which obscures the boundary of the cylindrical core and produces the smallest average size of the cylinder. Such a quasi-homogeneous binary solvent atmosphere perturbs the degree of order of the entire system and gives rise to the second highest value of the entropy and the lowest free energy density, as shown in Figure 5b. With a continuous increase in hydrophobic solvent to a significant amount, taking the system doped with 90% hydrophobic solvent of the total solvent shown in Figure 5c, as an example, the lowest content of hydrophilic solvent beads collected around the cylindrical core forms a negligible hydrophilic solvent shell with very low density, which has a slight influence on the self-assembly of the copolymer. In contrast, with the highest number of hydrophobic solvent beads, it is quite easy to form a hydrophobic solvent shell through the interior of the cylindrical core, and the intrusion and occupation of the hydrophobic solvent beads in the core domain push the hydrophobic branches of the copolymer aside, causing an evident decrease in the density of the MMA component. Then, a series of related responses from this pushing effect of the solvent beads causes an expanded arrangement of the copolymers, and, accordingly, an increase in the average size of the cylinder emerges. Owing to the highly ordered arrangement of the copolymer and the solvent, this system presents a lower entropy. Moreover, the comparable difference in the repulsion of the hydrophobic solvent beads with the EO and MMA components of the copolymer, in conjunction with the increased hydrophobic solvent content, synergistically promote the formation of a thicker hydrophobic solvent shell with a higher density, facilitating an increase in enthalpy. The second highest entropy in the system doped with 40% hydrophobic solvent and the resultant lowest free energy density both indicate that the system doped with nearly equal contents of the binary solvent favors generating the most thermodynamically stable system.

### 3.3. Self-Assembled Structure Under the Confinement

The surface properties of the confinement are vital parameters that should be considered, which can be realized via the adjustment of the interaction between the confinement and the components of the copolymer and the properties of the polymers under confinement. According to the morphology, the dimensionality is also a crucial parameter for characterizing the confinement, which is classified into one-dimensional (1D) [49,63,64,65,66], two-dimensional (2D) [67,68], and three-dimensional (3D) [69,70,71] confinement. In general, these types of confinement present in the forms of two confining walls, concentric rings and spheres, respectively. Due to the progressive change from 1D to 2D to 3D in the geometric construction of the confinement, there is a certain commonality in the relationship among the resultant self-assembled structures. Therefore, confinement is of great significance to our study of 1D-confined structures, and the results can be reasonably inferred as fundamental in the understanding of further advanced 2D- and 3D-confined structures.

To simplify the adopted 1D confinement, the confinements we designed are neutral, and the walls exert the same repulsive force on the A and B beads, i.e., *E*_wall-A_ = *E*_wall-B_ = 5.0 kJ·mol^−1^, indicating nonpreferential wetting for both copolymeric components. In addition, according to the different locations of the walls in the *L_x_* × *L_y_* × *L_z_* spatial lattice, two types of confinement are established: one is referred to as YW55 confinement, in which the walls are placed in the XZ plane, and the other is called ZW55 confinement, in which the walls are in the XY plane. The other parameter settings are identical to those in the previous sections.

**Figure 6:**
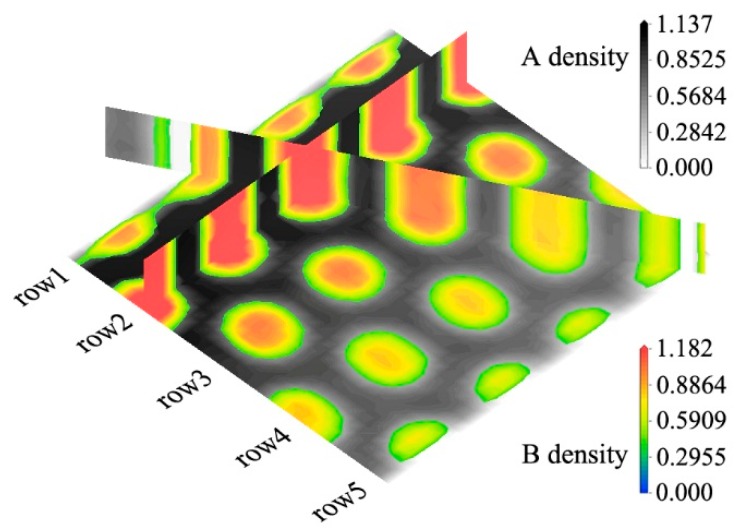
An equilibrium mesoscopic structure under YW55 confinement is extracted and specified by three characteristic density slices, with one along the X–Y plane, another through all cylindrical axes in row 2, and the third traversing individual cylindrical axes from every row. The color mappings of A and B beads are in white-black and reverse rainbow, respectively.

The Y-shaped copolymer self-assembles into five rows of cylindrical structures under YW55 confinement, as shown in Figure 6. In contrast to the uniformly assembled cylinders in the plain system, only the cylinders in the same row (i.e., parallel to the wall) are identical, whereas cylinders in different rows (i.e., perpendicular to the wall) are diverse in both volumetric size and radial density, which results from the repulsive force of the walls. The identical repulsive interaction force from both walls on the polymeric components provides the possibility of both A and B beads near both walls being equal, as well as the aggregation of the same component, so two featured semicylindrical arrays are generated as row 1 and row 5, respectively, which are strikingly different from the two completely cylindrical arrays in the plain system without confinement. The repulsion force from the walls repelling both A and B beads away from both walls results in fewer A and B beads remaining near the walls but makes them concentrated. Thus, the size of the aggregates near both walls (i.e. in row 1 and row 5) is the smallest compared with the sizes of other aggregates far from both walls (i.e., in row 2, row 3 and row 4). Here, the flat conformations of individual preferred blocks and nonpreferred blocks on the substrate surface has been demonstrated by a suite of surface-sensitive experiments [72], and their observation can support our simulation results of confined self-assembled structures.

**Figure 7:**
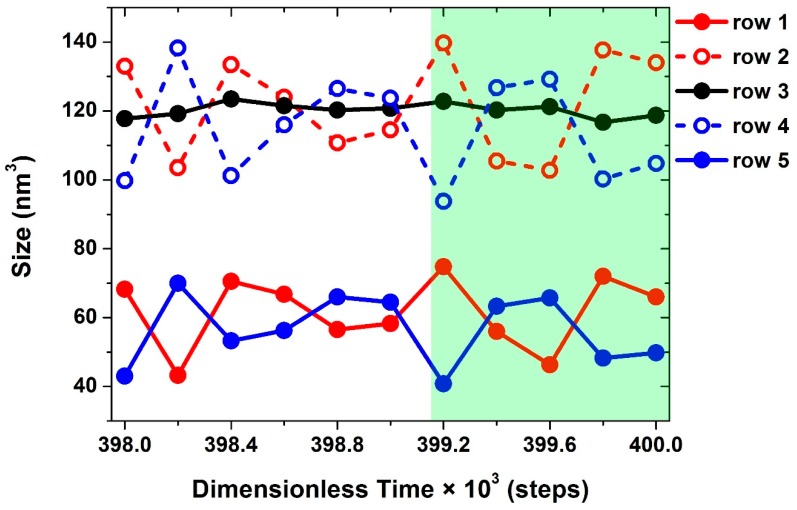
Under YW55 confinement, the average size of the cylinders in each row with the time evolving from 398.0 × 10^3^ steps to 400 × 10^3^ steps. The green region is selected for the following analysis given in Figure 8.

**Figure 8:**
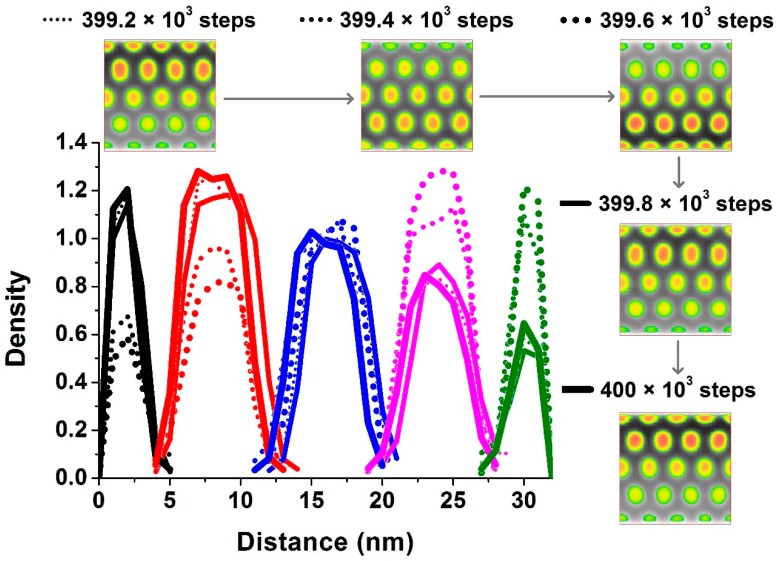
Density distributions of B beads in the radial direction under YW55 confinement at successive simulation time. The three dotted lines with increased widths correspond to the density at 399.2 × 10^3^ steps, 399.4 × 10^3^ steps and 399.6 × 10^3^ steps, and the two solid lines with increased widths correspond to the density at 399.8 × 10^3^ steps and 400 × 10^3^ steps. The density distributions of five individual cylinders from row 1 to row 5 are colored in black, red, blue, magenta and dark green, respectively.

In contrast, the numerous A and B beads in the middle region of the simulation box assemble into larger-sized cylinders. In the direction perpendicular to the walls, dynamic tracking of the average size in each row and the density distribution of the same string consisting of one selected cylinder from each row exhibit their differences explicitly and quantifiably. The curves of volumetric size for the cylinders from row 1, row 2, row 4 and row 5 present a periodically changing pattern until the self-assembly structure reaches a thermodynamics state, and the final three periodical changes are displayed in Figure 7. Additionally, the density distribution of the last period (i.e., a green colored zone from 399.2 × 10^3^ steps to 400 × 10^3^ steps in Figure 7) is extracted as a representative of the periodically changing pattern to be analyzed, as shown in Figure 8. Combining the results from Figure 7 and Figure 8, we see that the cylinders near the same side of a wall exhibit quite similar variation trends. Hence, three featured regions are distinguishable. The region near one wall including row 1 and row 2 is referred to as Region 1, the region in the middle including row 3 is referred to as Region 2, and the region near the other wall including row 4 and row 5 is referred to as Region 3. Among them, the cylinders from Region 1 and Region 3 are located near the walls, so an instant response of the copolymeric molecules to the repulsion from the walls is quite remarkable, resulting in the remarkably large variation in both size and density with time. However, the cylinders from Region 2 located in the middle region of the simulation box and are also far from both walls, so all the copolymeric molecules in this region experience the least repulsion from both walls, which gives rise to no significant change in either size or density. Corresponding to the qualitative analysis above, the statistical data for the positions of the cylindrical cores of the five rows over time is listed in Table 3. The highest quantitative standard deviation comes from row 3, which reveals that the range of motion of the cylinders in the middle region is the largest. The standard deviations for rows 2 and 4 are similar and nearly half of that for row 3, which indicates that the second-largest range of motion of the cylinders is from the rows second-nearest the walls. The standard deviations for row 1 and row 5 are very close, and both are lowest in value, being approximately 10% of the highest value from row 3, which indicates that the smallest range of motion of the cylinders is located nearest the walls.

Copolymeric molecules under YW55 confinement suffer two repulsive forces from the opposite direction from the two walls, which causes the density distribution to present periodic fluctuation. Thus, the self-assembly mechanism under YW55 confinement can be elaborated entirely in one periodic change. Here, we take the last periodic change, of which the time is from 399.2 × 10^3^ steps to 400 × 10^3^ steps, as the analyzed time range. The time of 399.2 × 10^3^ steps is assumed as the starting point, at which point the corresponding assembled structure results from the previous periodic change after several cycles. From the density slice at 399.2 × 10^3^ steps, we can see that the cylinders in row 1 and row 2 stay relatively farther from the top wall, favoring the copolymers adopting a relatively expanded conformation. Furthermore, the collected copolymers in this region aggregate into relatively larger sizes with relatively denser cores. Conversely, cylinders in row 4 and row 5 stay near the bottom wall, and the strong repulsion from this wall compels the EO and MMA segments to adopt a contracted conformation, and the copolymers flow away from this region, producing relatively smaller sizes of cylinders with relatively sparse cores. Therefore, from the quantitative analysis of the density distribution of B beads, we can see that the density of the cylindrical core in row 1 is higher than that in row 5, and in the same manner, the density of cylindrical core in row 2 is higher than that in row 4.

For the cylinders in row 3, if they are located in the middle region far from both walls, where the two repulsive forces from opposite directions are offset, and the cylinders are left in an environment without external effects, there will be no significant change in the size or density distribution for Region 2. However, at 399.2 × 10^3^ steps, the cylinders in row 3 remain relatively close to the bottom wall, which makes the cylinders suffer a weak net repulsive force, so a slight change appears in their size and density distribution. Similarly, such unbalanced force on the cylinders in different rows triggers their movement towards the top wall to different extents. Under direct and strong repulsion by the walls, the cylinders in rows 1 and 5 are confined within a narrow region with the smallest range of motion. Cylinders in rows 2 and 4 are located farther from the corresponding wall than are those in rows 1 and row 5, respectively, and the relatively weaker repulsion weakens the restriction on their mobility, suggesting a relatively larger range of motion. Due to the long distance from both walls, the cylinders in row 3 are free from the repulsion of both walls, facilitating the largest range of motion. The resulting inverted force from opposite walls between the cylinders in rows 1 and 5 and between the cylinders in rows 2 and 4 produces an inverted change trend between the size and density at 399.4 × 10^3^ steps. This is also the reason why the change tendencies for the size and density distribution of Region 1 and Region 3 are quite different. The former large change in the position of all cylinders towards the wall near row 1 pushes the system to recover in the following 0.4 × 10^3^ steps, until the time of 399.8 × 10^3^ steps, and then a new periodic change is activated. It can be inferred that the introduction of confinement, especially confinement with strong interaction with the copolymeric molecules, has the ability to bring the system into a kinetic equilibrium state.

### 3.4. Self-Assembled Structure Under Confinement Doped with a Single Solvent

**Figure 9:**
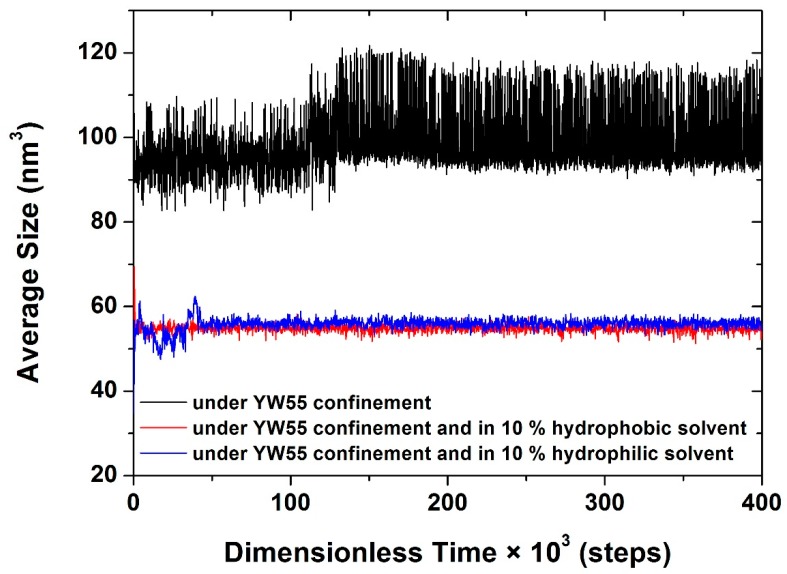
Average volumetric size of the self-assembled cylinders in three systems with time. They are the system under YW55 confinement denoted by the black line, the system under YW55 confinement and doped with 10% hydrophobic solvent denoted by the red line, and the system under YW55 confinement and doped with 10% hydrophilic solvent denoted by the blue line.

By comparison with the confined self-assembly, the same amount of 10% hydrophobic or hydrophilic single solvent is doped into the copolymer system to study the solvent effect on confined self-assembly, and the average size evolutions are shown in Figure 9. Under YW55 confinement, 129 × 10^3^ steps are required to regulate the smaller-sized cylinders to coalesce into larger cylinders. As we know from the former analysis of the confined self-assembly mechanism, due to the repulsion from the walls, the self-assembled cylinders are formed in hierarchical sizes along the direction perpendicular to the walls, presenting a broad amplitude change in the average size of all cylinders. The most striking change after the introduction of the solvent is the presence of a narrow amplitude change pattern instead of the broad pattern, indicating a uniformly sized self-assembled cylindrical structure. Another remarkable change is the dramatic decrease in the size adjustment stage from 129 × 10^3^ steps (YW55 confinement needs) to 10.2 × 10^3^ steps (doped with hydrophobic solvent) and 42.6 × 10^3^ steps (doped with hydrophilic solvent), respectively.

Benefiting from their small size, solvent beads disperse quickly and easily into the system and instantaneously take part in self-assembly. The presence of solvent beads dilutes the copolymer system and then reduces the gathering probability of the same copolymeric components, so these diluted copolymeric components assemble into small aggregates. When the copolymer system is doped with 10% hydrophobic solvent, the small solvent beads occupy both innermost regions near the two walls and form a thin solvent layer to weaken the repulsion between the walls and the internal system. Additionally, the solvent beads also concentrate within the domain rich in hydrophobic segments (i.e., the core domain of the cylinders), which share partial repulsion from the wall with the inner copolymeric components of the cylinders, and the entered solvent beads mixing with the hydrophobic segments does not hinder the following stage of size adjustment, resulting in the time of this stage being only 7.9% of that of the situation without any solvent. Similarly, the doped hydrophilic solvent beads engage in the aggregate domain rich in hydrophilic segments (i.e., the corona domain of the cylinders) and can also play a role in undertaking partial repulsion from the walls to avoid the formation of hierarchically sized cylinders. Compared with the formation of the dense core doped with hydrophobic solvent, it requires more time to open such a dense packing of the outer parts of the cylinders in the further coalescing of smaller aggregates or in the splitting of larger aggregates. Consequently, at the size adjustment stage, the system experiences six “coalescence-split” cycles until the system reaches a thermodynamically and kinetically stable state. Finally, the equilibrium average size of the confined self-assembled cylinders doped with the hydrophilic solvent is slightly greater than that of those doped with the hydrophobic solvent, so this difference in size can almost be ignored.

In contrast to YW55 confinement, ZW55 confinement possesses a shorter acting distance of 5 nm, which is only 15.625% of the former, and possesses a larger influencing area of 1024 nm^3^, which is 40.96-fold larger than that of the former. Both outstanding features of ZW55 confinement create a strong repulsion interaction environment to influence the self-assembly of the copolymeric system, which is beneficial for segregating the copolymers into small aggregates at the beginning. Assisted by the narrow space between the two walls, whose strong repulsion of the copolymeric component forces the hydrophobic segments and hydrophilic segments to tilt towards the wall, the conformations of copolymers are further refined via self-assembly to form a micellar structure. Driven by the high surface free energy, small micelles coalesce into larger micelles over the following 4.0 × 10^3^ steps, and then, the newly born larger micelles are quickly manipulated into a regular hexagonal arrangement until reaching a stable thermodynamic state. Differently from the resultant self-assembly under YW55 confinement, the copolymers self-assemble into a spherical micellar structure instead of a cylindrical structure, the size adjustment time sharply reduces to only 3.1%, and the equilibrium sizes of the micelles are uniform rather than hierarchical, decreasing approximately 57% in average size.

**Figure 10:**
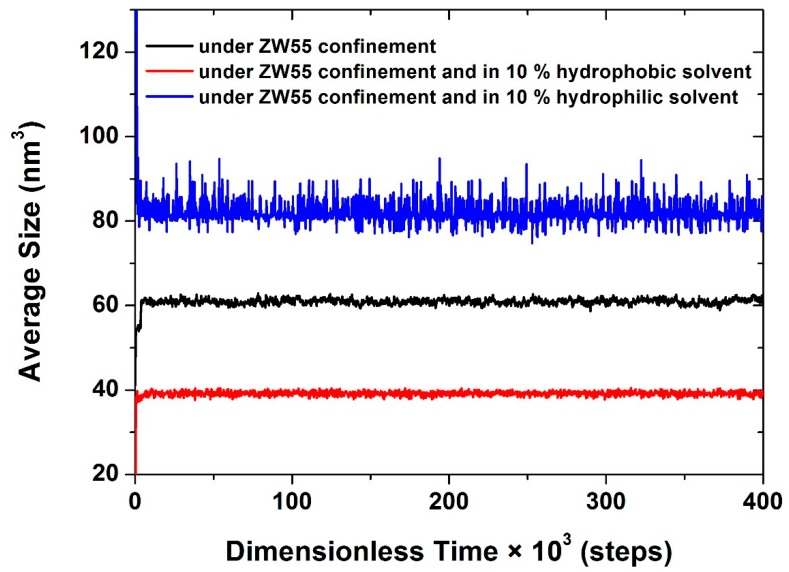
Average size of the self-assembled micelles in three systems over time. The system under ZW55 confinement is denoted by the black line, the system under ZW55 confinement and doped with 10% hydrophobic solvent is denoted by the red line, the system under ZW55 confinement and doped with 10% hydrophilic solvent is denoted by the blue line.

With the addition of solvent into a ZW55 confined system, the self-assembly mechanism of the copolymer system becomes remarkably different, as shown in Figure 10, and is closely related to the hydrophilicity of the solvent. When the doped solvent is hydrophobic, the solvent beads disperse into the system promptly and momentarily converge in the hydrophobic concentration domain. The invasion of solvent beads dilutes the density of the hydrophobic domain, and thus, more segregated hydrophobic aggregates are generated. Under the assistance of strong repulsion from the walls, the fast coalescence from the small micelles into the larger-sized micelles is completed in only 1.0 × 10^3^ steps, which presents the same “coalescence” mechanism as the situation with only ZW55 confinement, but the resultant number of micelles is twice as large, owing to the diluted hydrophobic cores being unfavorable for further coalescence. In contrast to the hydrophobic solvent, the hydrophilic solvent beads disperse into the hydrophilic concentrating domain, and the diluted continuous phase contributes little to the initial segregation of copolymer system, leading to the production of bulky aggregates. Subsequently, the bulky aggregates are split into small aggregates in only 1.4 × 10^3^ steps, presented as a “split” mechanism. Such a sparse density of the outer hydrophilic shells endows more degrees of freedom through which the inside hydrophobic segments can relax their conformations, facilitating the formation of a more stretched conformation and leading to loosely bound micelles with a larger average size of approximately 82 nm^3^. The packing of the small hydrophilic solvent beads in the outer shells of the micelles resists partial repulsion from the walls, which is unfavorable for generating uniformly sized micelles. Therefore, during the long equilibrium stage, the resultant average size of the micelles fluctuates with a large amplitude of change between 74 nm^3^ to 94 nm^3^ as time evolves.

## 4. Conclusions

The self-assembled structures of the Y-shaped copolymer system fall on the mesoscopic scale and were studied via computer modeling. The MesoDyn simulation method was adequate to study such topic, so it was adopted in this research. The plain copolymer system self-assembles into a cylindrical structure with a uniform size and density distribution. The introduction of solvent breaks the uniform distribution at 10%-doping levels of both hydrophobic and hydrophilic solvents, resulting from the access of the overwhelming hydrophobic solvent beads to the core domain with a high and widespread density, which remarkably disturbs and dilutes the density distribution of the self-assembled cylinders. Meanwhile, the entrance of the hydrophobic solvent beads promotes an expansion in the size of the cylinders. In contrast, the hydrophilic solvent beads disperse into the extensive hydrophilic gathering domain (located in the outer part of the cylinders). Its occupation and the repulsion facilitate the solvent beads forcing the hydrophobic domain of the copolymers to contract inward, resulting in a reduction of the average size of the cylinders. The shrunken conformation increases the density of the hydrophobic core but is weakened by doping with a small number of solvent beads, which decreases the density of the hydrophobic core, and both factors synergistically bring about a disturbance in the density distribution of the cylinders. Our theoretical results are consistent with the experimental results, in which different choices of solvent vapors offer different controlling abilities to a width of well-ordered features [33].

According to the diverse effects of different individual solvents on the self-assembly of the amphiphilic copolymer, further study of the effect of a mixture of hydrophobic and hydrophilic solvents on the same copolymeric system was performed. The self-assembled cylindrical structure is maintained when doped with a binary solvent, but the average size of the cylinders and the thermodynamic data of the system are distinguishable for different doping amounts of the hydrophobic solvent. The enthalpy increases with the increase in the hydrophobic solvent ratio, and a similar change occurs regarding the average size. However, the system doped with a nearly equal proportion of binary solvents (i.e., 40% hydrophobic solvent mixed with 60% hydrophilic solvent) possesses the lowest free energy density due to the second-highest entropy generated by the combined effect from the inner and outer gathering of the hydrophobic and hydrophilic solvent beads, respectively.

Under a weak repulsive environment such as that of YW55 confinement, with a long acting distance and a small influencing area, three apparent regions can be recognized from the trends in the change of the average size of cylinders in each row and the corresponding density distribution, including two regions adjacent to the individual wall and the middle region among the two walls. Notably, over time, the average size and density distributions of the cylinders in the middle region remain unchanged, and these cylinders present the largest range of motion compared with the movement of the cylinders in the former two regions. On the other hand, when the average size and density distributions of the cylinders in one region adjacent to one wall decrease, the average size and density distributions of the cylinders in the other region adjacent to the other wall increase accordingly. Additionally, the cylinders closest to the walls, being restricted to the smallest range of motion, show the greatest amplitude of variation. Consequently, apparently hierarchically sized cylinders are generated during such a dynamic equilibrium process resulting from confinement. It is amazing that with the doping of a hydrophobic solvent or hydrophilic solvent into the YW55-confined copolymer system, the resulting uniformly sized cylinders replace the hierarchically sized cylinders due to participation in the hydrophobic solvent gathered in the region composed of the cylindrical core or the hydrophilic solvent collected in the region constituted by the cylindrical shell. Such a distribution of hydrophobic or hydrophilic solvent beads in the self-assembled cylinders offsets the repulsion from the walls and helps to construct a similar unperturbed external environment, so that the final average sizes under these two solvent environments are quite close.

When YW55 confinement is replaced by ZW55 confinement, which is a strong repulsive environment consisting of a short acting distance and a large influencing region, the copolymers self-assemble into a spherical micellar structure instead of a cylindrical structure. The small hydrophobic solvent beads are compatible with the hydrophobic segments of the copolymer and are assisted by strong repulsion from the walls, and many small aggregates consisting of not only the mixture with copolymeric hydrophobic segments and solvent beads inside but also those with copolymeric hydrophilic segments outside are generated. The subsequent coalescence of small aggregates is easily realized via the opening of the outer shells consisting of only copolymeric hydrophilic segments. Due to the diluted density of the inner micellar core, it is relatively difficult to collect hydrophobic segments, resulting in micelles that are smaller than average and uniform in size. A “coalescence” mechanism for the hydrophobic solvent involved in the self-assembly is elaborated. In contrast, hydrophilic solvent beads are mixed in the continuous phase of the copolymeric hydrophilic gathering domain, which is unfavorable to the formation of segregation. The subsequent decrease in entropy drives the dissociation of large aggregates. Assisted by the diluted outside hydrophilic shells, hydrophobic segments in the individual micelles are favorable for expanding outwards to different extents. A “splitting” mechanism for the hydrophilic solvent presents in this self-assembly, generating a larger average size but an undulating change in the average size. 

Our results provide solid and abundant theoretical evidence of a solvent effect on the self-assembly of copolymer systems, which may point to a new perspective on the manufacture of nanomaterials with the aid of solvents. The ability of hydrophilic/hydrophobic solvent entering and gathering in the hydrophilic/hydrophobic domain of the self-assembly can be utilized to control the aggregation structure of self-assembly, further, to realize the regulation of its function, which can be potentially applied to manufacture chiral separation membranes, drug release systems, antifouling coatings, gas and biosensors, activating carriers for biocatalysts and so on. 

## Figures and Tables

**Table 1:** Input parameters (*E*_ij_) information.

*E*_ij_ (kJ·mol^−1^)	A	B	Hydrophilic Solvent	Hydrophobic Solvent
A	0	4.68	1.0	5.0
B	4.68	0	5.0	3.0
hydrophilic solvent	1.0	5.0	0	6.0
hydrophobic solvent	5.0	3.0	6.0	0

**Table 2:** Statistics of five peak values in the density distribution of B beads originating from Figure 2.

System	Average Value	Standard Deviation
(a) plain copolymer	1.044	0.022
(b) copolymer doped with 10% hydrophobic solvent	0.864	0.080
(c) copolymer doped with 10% hydrophilic solvent	0.958	0.039

**Table 3:** The position statistics of the five cores originating from Figure 8.

Index	Average Position of Cores on the Z-axis (nm)	Standard Deviation
row 1	1.679	0.064
row 2	8.336	0.436
row 3	16.188	0.612
row 4	23.889	0.387
row 5	30.372	0.062

## References

[1] Reiter G., Al Akhrass S., Hamieh M., Damman P., Gabriele S., Vilmin T., Raphaël E. (2009). Dewetting as an investigative tool for studying properties of thin polymer films. Eur. Phys. J. Spec. Top..

[2] Muñoz-Bonilla A., Fernández-García M., Rodríguez-Hernández J. (2014). Towards hierarchically ordered functional porous polymeric surfaces prepared by the breath figures approach. Prog. Polym. Sci..

[3] Morishima Y., Nomura S., Ikeda T., Seki M., Kamachi M. (1995). Characterization of unimolecular micelles of random copolymers of sodium 2-(acrylamido)-2-methylpropanesulfonate and methacrylamides bearing bulky hydrophobic substituents. Macromolecules.

[4] Rösler A., Vandermeulen G.W.M., Klok H.A. (2012). Advanced drug delivery devices via self-assembly of amphiphilic block copolymers. Adv. Drug Deliv. Rev..

[5] Kylian O., Shelemin A., Solar P., Choukourov A., Hanus J., Vaidulych M., Kuzminova A., Biederman H. (2017). Plasma polymers: From thin films to nanocolumnar coatings. Thin Solid Films.

[6] Bormashenko E., Pogreb R., Stanevsky O., Bormashenko Y., Tamir S., Cohen R., Nunberg M., Gaisin V.-Z., Gorelik M., Gendelman O.V. (2005). Mesoscopic and submicroscopic patterning in thin polymer films: Impact of the solvent. Mater. Lett..

[7] Cohen E., Weissman H., Pinkas I., Shimoni E., Rehak P., Král P., Rybtchinski B. (2018). Controlled self-assembly of photofunctional supramolecular nanotubes. ACS Nano.

[8] Dwivedi S., Mukherjee V.R., Atta A. (2017). Formation and control of secondary nanostructures in electro-hydrodynamic patterning of ultra-thin films. Thin Solid Films.

[9] Bernardin G.A., Davies N.A., Finlayson C.E. (2017). Spray-coating deposition techniques for polymeric semiconductor blends. Mater. Sci. Semicond. Proc..

[10] Credi C., Pintossi D., Bianchi C.L., Levi M., Griffini G., Turri S. (2017). Combining stereolithography and replica molding: On the way to superhydrophobic polymeric devices for photovoltaics. Mater. Des..

[11] Chopra A.M., Mehta M., Bismuth J., Shapiro M., Fishbein M.C., Bridges A.G., Vinters H.V. (2017). Polymer coating embolism from intravascular medical devices—A clinical literature review. Cardiovasc. Pathol..

[12] Crossland E.J.W., Kamperman M., Nedelcu M., Ducati C., Wiesner U., Smilgies D.-M., Toombes G.E.S., Hillmyer M.A., Ludwigs S., Steiner U. (2009). A bicontinuous double gyroid hybrid solar cell. Nano Lett..

[13] Park S., Wang J.Y., Kim B., Xu J., Russell T.P. (2008). A simple route to highly oriented and ordered nanoporous block copolymer templates. ACS Nano.

[14] Lee B., Yoon J., Oh W., Hwang Y., Heo K., Jin K.S., Kim J., Kim K.W., Ree M. (2005). In-situ grazing incidence small-angle X-ray scattering studies on nanopore evolution in low-*k* organosilicate dielectric thin films. Macromolecules.

[15] Sidorenko A., Tokarev I., Minko S., Stamm M. (2003). Ordered reactive nanomembranes/nanotemplates from thin films of block copolymer supramolecular assembly. J. Am. Chem. Soc..

[16] Park S., Lee D.H., Xu J., Kim B., Hong S.W., Jeong U., Xu T., Russell T.P. (2009). Macroscopic 10-terabit-per-square-inch arrays from block copolymers with lateral order. Science.

[17] Gersappe D., Fasolka M., Israels R., Balazs A.C. (1995). Modeling the behavior of random copolymer brushes. Macromolecules.

[18] Zhulina E.B., Singh C., Balazs A.C. (1996). Forming patterned films with tethered diblock copolymers. Macromolecules.

[19] Ferreira P.G., Leibler L. (1996). Copolymer brushes. J. Chem. Phys..

[20] Brown G., Chakrabarti A., Marko J.F. (1996). Layering phase separation of densely grafted diblock copolymers. Macromolecules.

[21] Wang J., Müller M. (2009). Microphase separation of diblock copolymer brushes in selective solvents: Single-chain-in-Mean-Field simulations and integral geometry analysis. Macromolecules.

[22] Akgun B., Ugur G., Brittain W.J., Majkrzak C.F., Li X., Wang J., Li H., Wu D.T., Wang Q., Foster M.D. (2009). Internal structure of ultrathin diblock copolymer brushes. Macromolecules.

[23] Guskova O.A., Seidel C. (2011). Mesoscopic simulations of morphological transitions of stimuli-responsive diblock copolymer brushes. Macromolecules.

[24] Rudov A.A., Khalatur P.G., Potemkin I.I. (2012). Perpendicular domain orientation in dense planar brushes of diblock copolymers. Macromolecules.

[25] Tobis J., Boch L., Thomann Y., Tiller J.C. (2011). Amphiphilic polymer conetworks as chiral separation membranes. J. Membr. Sci..

[26] Lin C., Gitsov I. (2010). Preparation and characterization of novel amphiphilic hydrogels with covalently attached drugs and fluorescent markers. Macromolecules.

[27] Gudipati C.S., Greenlief C.M., Johnson J.A., Prayongpan P., Wooley K.L. (2004). Hyperbranched fluoropolymer and linear poly(ethylene glycol) based amphiphilic crosslinked networks as efficient antifouling coatings: An insight into the surface compositions, topographies, and morphologies. J. Polym. Sci. Pol. Chem..

[28] Hanko M., Bruns N., Rentmeister S., Tiller J.C., Heinze J. (2006). Nanophase-separated amphiphilic conetworks as versatile matrixes for optical chemical and biochemical sensors. Anal. Chem..

[29] Savin G., Bruns N., Thomann Y., Tiller J.C. (2005). Nanophase separated amphiphilic microbeads. Macromolecules.

[30] Bodycomb J., Funaki Y., Kimishima K., Hashimoto T. (1999). Single-grain lamellar microdomain from a diblock copolymer. Macromolecules.

[31] Morkved T.L., Lu M., Urbas A.M., Ehrichs E.E., Jaeger H.M., Mansky P., Russell T.P. (1996). Local control of microdomain orientation in diblock copolymer thin films with electric fields. Science.

[32] Matsen M.W. (2005). Stability of a block-copolymer lamella in a strong electric field. Phys. Rev. Lett..

[33] Jeong J.W., Park W.I., Kim M.-J., Ross C.A., Jung Y.S. (2011). Highly tunable self-assembled nanostructures from a poly(2-vinylpyridine-*b*-dimethylsiloxane) block copolymer. Nano Lett..

[34] Mu D., Li J.Q., Wang S. (2011). Modeling and analysis of the compatibility of poly(ethylene oxide)/poly(methyl methacrylate) blends with surface and shear inducing effects. J. Appl. Polym. Sci..

[35] Mu D., Li J.Q., Zhou Y.H. (2011). Modeling and analysis of the compatibility of polystyrene/poly(methyl methacrylate) blends with four inducing effects. J. Mol. Model..

[36] Mu D., Li J.Q., Wang S. (2012). Mesoscopic simulation of the surface inducing effects on the compatibility of PS-*b*-PMMA copolymers. J. Appl. Polym. Sci..

[37] Kim S., Shin D.O., Choi D.-G., Jeong J.-R., Mun J.H., Yang Y.-B., Kim J.U., Kim S.O., Jeong J.-H. (2012). Graphoepitaxy of block-copolymer self-assembly integrated with single-step ZnO nanoimprinting. Small.

[38] Rockford L., Liu Y., Mansky P., Russell T.P., Yoon M., Mochrie S.G.J. (1999). Polymers on nanoperiodic, heterogeneous surfaces. Phys. Rev. Lett..

[39] Tavakkoli A., Gotrik K.G.K.W., Hannon A.F., Alexander-Katz A., Ross C.A., Berggren K.K. (2012). Templating three-dimensional self-Assembled structures in bilayer block copolymer films. Science.

[40] Yang X.M., Peters R.D., Nealey P.F., Solak H.H., Cerrina F. (2000). Guided self-assembly of symmetric diblock copolymer films on chemically nanopatterned substrates. Macromolecules.

[41] Park S.M., Craig G.S.W., La Y.H., Solak H.H., Nealey P.F. (2007). Square arrays of vertical cylinders of PS-*b*-PMMA on chemically nanopatterned surfaces. Macromolecules.

[42] Stoykovich M.P., Müller M., Kim S.O., Solak H.H., Edwards E.W., de Pablo J.J., Nealey P.F. (2005). Directed assembly of block copolymer blends into nonregular device-oriented structures. Science.

[43] Kim S.O., Solak H.H., Stoykovich M.P., Ferrier N.J., de Pablo J.J., Nealey P.F. (2003). Epitaxial self-assembly of block copolymers on lithographically defined nanopatterned substrates. Nature.

[44] Ruiz R., Kang H., Detcheverry F.A., Dobisz E., Kercher D.S., Albrecht T.R., de Pablo J.J., Nealey P.F. (2008). Density multiplication and improved lithography by directed block copolymer assembly. Science.

[45] Cheng J.Y., Rettner C.T., Sanders D.P., Kim H.-C., Hinsberg W.D. (2008). Dense self-assembly on sparse chemical patterns: Rectifying and multiplying lithographic patterns using block copolymers. Adv. Mater..

[46] Tada Y., Akasaka S., Yoshida H., Hasegawa H., Dobisz E., Kercher D., Takenaka M. (2008). Directed self-assembly of diblock copolymer thin films on chemically-patterned substrates for defect-free nano-patterning. Macromolecules.

[47] Chen P., Liang H., Xia R., Qian J., Feng X. (2013). Directed self-assembly of block copolymers on sparsely nanopatterned substrates. Macromolecules.

[48] Mu D., Li J.Q., Wang S. (2015). Changes in the phase morphology of miktoarm PS-*b*-PMMA copolymer induced by a monolayer surface. Colloid Polym. Sci..

[49] Mu D., Li J.Q., Feng S.Y. (2017). One-dimensional confinement effect on the self-assembly of symmetric H-shaped copolymers in a thin film. Sci. Rep..

[50] Yin Y., Jiang R., Li B., Jin Q., Ding D., Shi A.-C. (2008). Self-assembly of grafted Y-shaped ABC triblock copolymers in solutions. J. Chem. Phys..

[51] Sun J., Chen X., Guo J., Shi Q., Xie Z., Jing X. (2009). Synthesis and self-assembly of a novel Y-shaped copolymer with a helical polypeptide arm. Polymer.

[52] Dong R., Zhong Z., Hao J. (2012). Self-assembly of onion-like vesicles induced by charge and rheological properties in anionic-nonionic surfactant solutions. Soft Matter.

[53] Mu D., Li J.Q., Feng S.Y. (2015). Mesoscale simulation of the formation and dynamics of lipid-structured poly(ethylene oxide)-*block*-poly(methyl methacrylate) diblock copolymers. Phys. Chem. Chem. Phys..

[54] Mu D., Li J.Q., Feng S.Y. (2017). Self-assembled morphologies of an amphiphilic Y-shaped weak polyelectrolyte in a thin film. Phys. Chem. Chem. Phys..

[55] Fraaije J.G.E.M., van Vlimmeren B.A.C., Maurits N.M., Postma M., Evers O.A., Hoffmann C., Altevogt P., Goldbeck-Wood G. (1997). The dynamic mean-field density functional method and its application to the mesoscopic dynamics of quenched block copolymer melts. J. Chem. Phys..

[56] van Vlimmeren B.A.C., Maurits N.M., Zvelindovsky A.V., Sevink G.J.A., Fraaije J.G.E.M. (1999). Simulation of 3D mesoscale structure formation in concentrated aqueous solution of the triblock polymer surfactants (ethylene oxide)_13_(propylene oxide)_30_(ethylene oxide)_13_ and (propylene oxide)_19_(ethylene oxide)_33_(propylene oxide)_19_. Application of dynamic mean-field density functional theory. Macromolecules.

[57] Crank J., Nicolson P. (1947). A practical method for numerical evaluation of solutions of partial differential equations of the heat conduction type. Math. Proc. Camb. Philos. Soc..

[58] Mu D., Huang X.R., Lu Z.Y., Sun C.C. (2008). Computer simulation study on the compatibility of poly(ethylene oxide)/poly(methyl methacrylate) blends. Chem. Phys..

[59] Grulke E.A. (1999). Solubility Parameter Values in Polymer Handbook.

[60] Brandrup J., Immergut E.H., Grulke E.A. (1999). Polymer Handbook.

[61] Zhu J., Jiang W. (2005). Self-assembly of ABC triblock copolymer into giant segmented wormlike micelles in dilute solution. Macromolecules.

[62] Li Z., Kesselman E., Talmon Y., Hillmyer M.A., Lodge T.P. (2004). Multicompartment micelles from ABC miktoarm stars in water. Science.

[63] Mu D., Li J.Q., Feng S.Y. (2017). Mechanistic investigations of confinement effects on the self-assembly of symmetric amphiphilic copolymers in thin films. Phys. Chem. Chem. Phys..

[64] Mu D., Li J.Q., Feng S.Y. (2015). Morphology of lipid-like structured weak polyelectrolyte poly(ethylene oxide)-*block*-poly(methyl methacrylate) diblock copolymers induced by confinements. Soft Matter.

[65] Mu D., Li J.Q., Feng S.Y. (2015). Mesoscopic simulation of the self-assembly of the weak polyelectrolyte poly(ethylene oxide)-*block*-poly(methyl methacrylate) diblock copolymers. Soft Matter.

[66] Wang Q., Yan Q., Nealey P.F., de Pablo J.J. (2000). Monte Carlo simulations of diblock copolymer thin films confined between two homogeneous surfaces. J. Chem. Phys..

[67] Dobriyal P., Xiang H., Kazuyuki M., Chen J.T., Jinnai H., Russell T.P. (2009). Cylindrically confined diblock copolymers. Macromolecules.

[68] Wang Y., Qin Y., Berger A., Yau E., He C., Zhang L., Gösele U., Knez M., Steinhart M. (2009). Nanoscopic morphologies in block copolymer nanorods as templates for atomic-layer deposition of semiconductors. Adv. Mater..

[69] Rider D.A., Chen J.I.L., Eloi J.C., Arsenault A.C., Russell T.P., Ozin G.A., Manners I. (2008). Controlling the morphologies of organometallic block copolymers in the 3-dimensional spatial confinement of colloidal and inverse colloidal crystals. Macromolecules.

[70] Chen P., Liang H., Shi A.C. (2008). Microstructures of a cylinder-forming diblock copolymer under spherical confinement. Macromolecules.

[71] Li S., Chen P., Zhang L., Liang H. (2011). Geometric frustration phases of diblock copolymers in nanoparticles. Langmuir.

[72] Sen M., Jiang N., Endoh M.K., Koga T., Ribbe A., Rahman A., Kawaguchi D., Tanaka K., Smilgies D.-M. (2018). Locally favored two-dimensional structures of block copolymer melts on nonneutral surfaces. Macromolecules.

